# Quantification of amyloid PET for future clinical use: a state-of-the-art review

**DOI:** 10.1007/s00259-022-05784-y

**Published:** 2022-04-07

**Authors:** Hugh G. Pemberton, Lyduine E. Collij, Fiona Heeman, Ariane Bollack, Mahnaz Shekari, Gemma Salvadó, Isadora Lopes Alves, David Vallez Garcia, Mark Battle, Christopher Buckley, Andrew W. Stephens, Santiago Bullich, Valentina Garibotto, Frederik Barkhof, Juan Domingo Gispert, Gill Farrar, on behalf of the AMYPAD consortium

**Affiliations:** 1 GE Healthcare, Amersham, UK; 2 Centre for Medical Image Computing (CMIC), Department of Medical Physics and Bioengineering, University College London, London, UK; 3 UCL Queen Square Institute of Neurology, University College London, London, UK; 4 Department of Radiology and Nuclear Medicine, Amsterdam Neurocience, Amsterdam UMC, Vrije Universiteit Amsterdam, Amsterdam, The Netherlands; 5 Barcelonaβeta Brain Research Center (BBRC), Pasqual Maragall Foundation, Barcelona, Spain; 6 Universitat Pompeu Fabra, Barcelona, Spain; 7 IMIM (Hospital del Mar Medical Research Institute), Barcelona, Spain; 8 Clinical Memory Research Unit, Department of Clinical Sciences, Lund University, Malmö, Sweden; 9 Brain Research Center, Amsterdam, The Netherlands; 10 Life Molecular Imaging GmbH, Berlin, Germany; 11 Division of Nuclear Medicine and Molecular Imaging, University Hospitals of Geneva, Geneva, Switzerland; 12 NIMTLab, Faculty of Medicine, University of Geneva, Geneva, Switzerland; 13 Centro de Investigación Biomédica en Red Bioingeniería, Biomateriales y Nanomedicina, Madrid, Spain

**Keywords:** Brain, Amyloid, PET, Quantification, Alzheimer’s, Dementia, Centiloid, SUVr

## Abstract

Amyloid-β (Aβ) pathology is one of the earliest detectable brain changes in Alzheimer’s disease (AD) pathogenesis. The overall load and spatial distribution of brain Aβ can be determined in vivo using positron emission tomography (PET), for which three fluorine-18 labelled radiotracers have been approved for clinical use. In clinical practice, trained readers will categorise scans as either Aβ positive or negative, based on visual inspection. Diagnostic decisions are often based on these reads and patient selection for clinical trials is increasingly guided by amyloid status. However, tracer deposition in the grey matter as a function of amyloid load is an inherently continuous process, which is not sufficiently appreciated through binary cut-offs alone. State-of-the-art methods for amyloid PET quantification can generate tracer-independent measures of Aβ burden. Recent research has shown the ability of these quantitative measures to highlight pathological changes at the earliest stages of the AD *continuum* and generate more sensitive thresholds, as well as improving diagnostic confidence around established binary cut-offs. With the recent FDA approval of aducanumab and more candidate drugs on the horizon, early identification of amyloid burden using quantitative measures is critical for enrolling appropriate subjects to help establish the optimal window for therapeutic intervention and secondary prevention. In addition, quantitative amyloid measurements are used for treatment response monitoring in clinical trials. In clinical settings, large multi-centre studies have shown that amyloid PET results change both diagnosis and patient management and that quantification can accurately predict rates of cognitive decline. Whether these changes in management reflect an improvement in clinical outcomes is yet to be determined and further validation work is required to establish the utility of quantification for supporting treatment endpoint decisions. In this state-of-the-art review, several tools and measures available for amyloid PET quantification are summarised and discussed. Use of these methods is growing both clinically and in the research domain. Concurrently, there is a duty of care to the wider dementia community to increase visibility and understanding of these methods.

## Introduction

### Amyloid-β and the AD continuum

Alzheimer’s disease (AD) is the most common cause of dementia, accounting for 60–80% of cases above 65 years of age [1]. One of the earliest detectable brain changes in AD pathogenesis is amyloid-β (Aβ) plaque accumulation [2–4]. However, historically, AD has been diagnosed solely based on symptomatology, with a definite diagnosis only possible by *post-mortem* examination. With the recent arrival and increased availability of biomarkers for AD pathology, there has been a shift towards biomarker-based diagnosis, which can be appreciated in the 2007 research diagnosis criteria from the International Working Group [5, 6]. Updated in 2021 [7], the guidelines now further highlight amyloid’s central role in the AD diagnostic process. In research settings, a biomarker-only classification scheme has even been proposed, the amyloid/tau/neurodegeneration (A/T/N) framework [8], which further highlights the shift towards a biological definition of the disease independent of clinically defined diagnostic schemes. Detection of abnormal Aβ only, referred to as “Alzheimer’s pathologic change” (A+/T−), is considered the essential first step and if followed by a pathological change in tau progresses to the classification of AD (A+/T+) — with or without dementia. Amyloid biomarkers have been used as part of the A/T/N framework in large validation studies of population-based cohorts [9, 10], memory clinic populations [11, 12], cognitively unimpaired subjects [13], and longitudinal cognitive outcomes [9, 11, 13]. The central role of amyloid pathology across the AD *continuum* has been of major interest for both AD clinical research and drug development [14–17]. Alongside the development of cerebrospinal fluid (CSF) and blood-based biomarkers, molecular imaging using positron emission tomography (PET) plays an increasingly important role in determining biomarker status [18].

### Amyloid PET

**Fig. 1:**
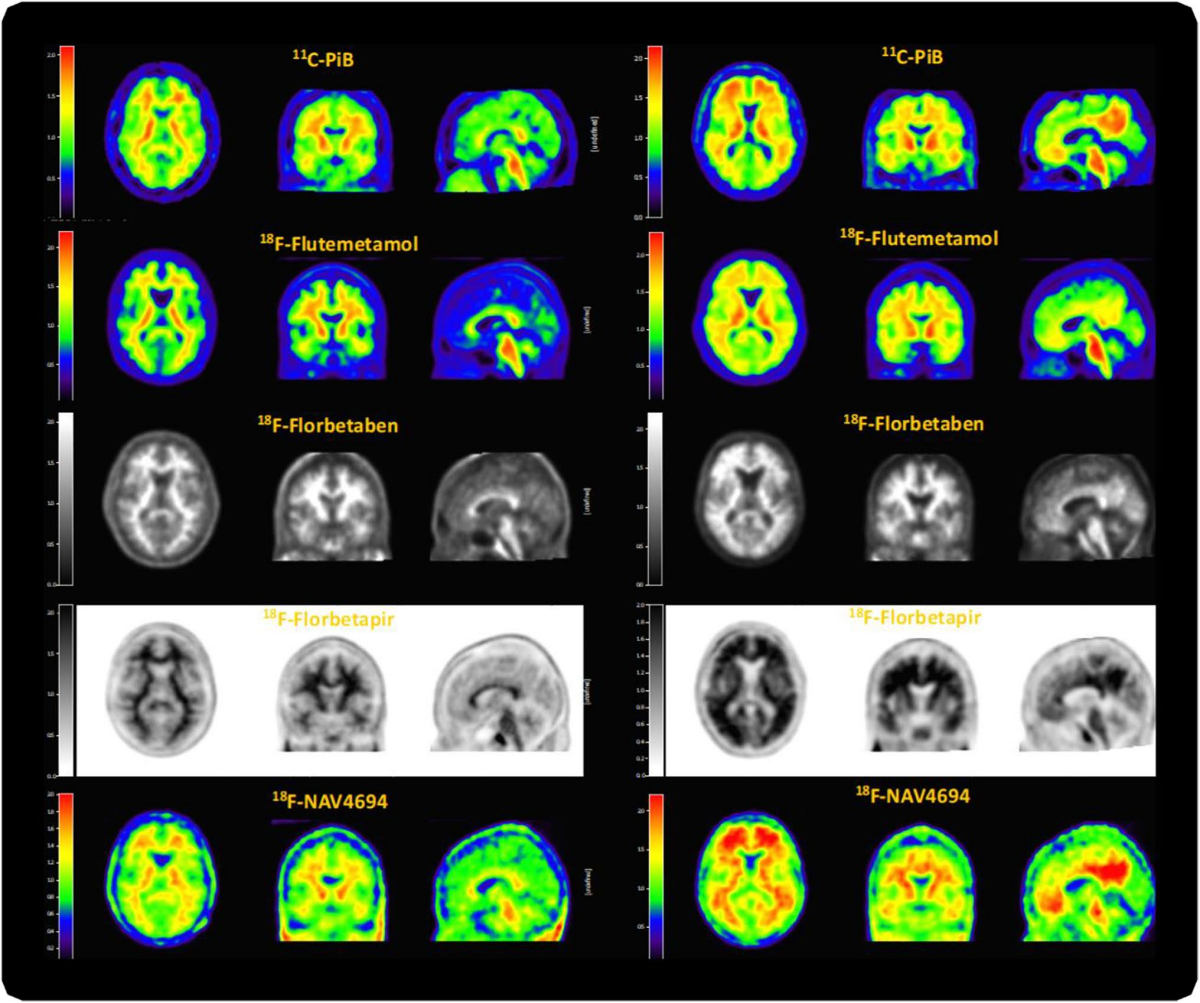
Illustrative PET images derived from the five most commonly used amyloid tracers on different patients. The left column shows Aß negative subjects (all ~0 Centiloid) and right column shows Aß positive subjects (all ~50 Centiloid, for further details, see “Centiloid scaling” section). Colour schemes used for regulatory approved tracers are in line with each of their FDA label prescribing information: [^18^F]flutemetamol (https://www.accessdata.fda.gov/drugsatfda_docs/label/2016/203137s005lbl.pdf), [^18^F]florbetaben (https://www.accessdata.fda.gov/drugsatfda_docs/label/2014/204677s000lbl.pdf), [^18^F]florbetapir (https://www.accessdata.fda.gov/drugsatfda_docs/label/2012/202008s000lbl.pdf)

The use of amyloid PET allows for the in vivo visualisation and quantification of Aβ protein fibrillary deposits, directly providing information on the total load and spatial distribution of Aβ pathology. Three fluorine-18 amyloid PET tracers are currently available for routine clinical use (Fig. 1) and have been validated against Consortium to Establish a Registry for Alzheimer’s Disease (CERAD) pathology as the gold standard. These radiotracers are [^18^F]florbetapir (Amyvid™; Avid Radiopharmaceuticals; approved in 2012) [19], [^18^F]flutemetamol (Vizamyl™; GE Healthcare; approved in 2013) [20], and [^18^F]florbetaben (Neuraceq™; Life Molecular Imaging; approved in 2014) [21]. Each of these radiotracers has different pharmacokinetics, chemical structure, and binding site/properties. However, they have all been approved by the Food and Drug Administration (FDA) and European Medicines Authority (EMA) for routine clinical use, and have local regulatory approval in other countries, such as Japan and Korea. The tracers are also widely used by the research community. In addition, other known compounds such as the carbon-11 labelled Pittsburgh compound B ([^11^C]PiB) [22] and [^18^F]NAV4694 [18, 23] are available for investigational use only.

### Clinical utility of amyloid PET

Routine clinical use of amyloid PET tracers involves visual assessment and binary categorisation of scans, based on tracer-specific manufacturers’ guidelines [24–26]. Classification is either *negative* (predominantly white matter uptake) or *positive* (binding in one or more cortical brain regions, or the striatum for [^18^F]flutemetamol and [^11^C]PiB). Certified readers are required by the regulatory authorities to complete and pass a training program specific to each radiotracer [27–29]. The visual assessment scales and guidelines are different for each radiotracer. However, high inter-rater agreement for visual rating protocols has been demonstrated for all ^18^F-labelled amyloid tracers [30], suggesting that visual interpretation of amyloid imaging by experts is not dependent on the rating protocol. Furthermore, efforts to create a universal visual assessment protocol for all amyloid imaging tracers are underway [31].

Over the past decade, many studies have demonstrated the level of analytical and clinical validity of amyloid PET in routine clinical practice [25, 29, 32–40]. More specifically, real-world studies have shown that disclosure of amyloid PET imaging results leads to a change in etiological diagnosis in approximately 25–31% of cases [33, 35, 36], significant increases in diagnostic confidence [33, 36, 38–40], and changes in patient management in approximately 37–72% of cases [35–38]. Appropriate use criteria have also been published for amyloid PET [41]. However, recent evidence suggests that patients beyond the appropriate use criteria can also benefit from amyloid PET through changes in management and diagnosis [42]. For example, research has suggested that anti-amyloid strategies could be a relevant approach to slow disease progression in Parkinson’s disease and Lewy body dementia [43, 44]. Also, in subjective cognitive decline (SCD) patients, for whom a positive or negative amyloid status can increase diagnostic confidence [33, 34, 37]. The largest clinical utility study to date is the Imaging Dementia-Evidence for Amyloid Scanning (IDEAS) study, which was designed to investigate the clinical utility of amyloid PET. The study enrolled over 18,000 patients from 946 dementia specialists at 595 centres in America [34]. Of the 11,409 patients completing study procedures, the composite endpoint changed in 4159 of 6905 patients with MCI (60.2%), the etiological diagnosis changed from AD to non-AD in 2860 (25.1%), and from non-AD to AD in 1201 (10.5%) cases, which was linked with changes in clinical management within 90 days. Whether these changes in management reflect an improvement in clinical outcomes for dementia patients is yet to be determined.

#### Global multi-centre studies adopting amyloid PET

Global multi-centre studies and consortia aiming to unravel the influence, prognostic value, and role of amyloid deposition in the AD timeline have been ongoing for some time. The Alzheimer’s Disease Neuroimaging Initiative (ADNI) study began in 2005 [45] and has acquired amyloid PET in thousands of mainly MCI patients [46–48] (http://adni.loni.usc.edu/). The first results from the Australian Imaging, Biomarkers and Lifestyle (AIBL) study were published in 2009 [49] and has continued to monitor over 1,000 volunteers (http://adni.loni.usc.edu/category/aibl-study-data/). More recently, in 2016, the AMYPAD consortium was initiated involving multiple academic and private research partners (https://amypad.eu/). AMYPAD consists of two substudies: (i) the diagnostic and patient management study (DPMS) [50], assessing amyloid PET’s impact on clinical management and diagnosis where quantitative measures will be the secondary endpoint; and (ii) the prognostic and natural history study (PNHS) [51]. In the PNHS, quantitative measures are the primary endpoint and amyloid PET is used to understand the development of AD in the pre-dementia phase of the disease, including cognitively unimpaired, SCD, and MCI participants. Given these goals, another major objective of AMYPAD is the development and validation of robust standardised methodology for quantitatively measuring brain amyloid [52], see “Future directions” section later in this review for an overview of AMYPAD’s ongoing studies. Studies such as these highlight the importance of amyloid PET and quantitative measures across the AD continuum, while visual reading remains the most common method of Aβ pathology in clinical routine.

#### Challenges of amyloid PET visual assessment across the clinical spectrum

Phase III autopsy validation studies have shown that binary classification through visual assessment is approximately 90% accurate in advanced clinical and end-of-life subjects, providing a useful stratification of Aβ status for clinical routine, clinical trials, and research purposes [20, 21, 27]. In a heterogeneous clinical population, visual assessment can be challenged by partial volume effects compounded by cortical thinning or atrophy, which in turn raises the question of whether or not to perform partial volume correction (PVC). The field remains divided on this issue, where recent evidence suggests that PVC can increase sensitivity for detecting early stage cerebral amyloidosis [53], but other studies comparing techniques have proven inconclusive [54, 55]. In addition, comorbidities such as normal pressure hydrocephalus [56] or other neurodegenerative disorders can further complicate visual assessments [29, 57–60]. However, the proportion of pre-dementia patients assessed in memory clinics has significantly increased over the past few years, with up to ~25% of patients presenting with SCD [61]. In these subjects, amyloid deposition may be emerging or focal [62], which makes visual assessment more challenging, especially by less experienced readers [63]. In such cases, the dichotomous approach is more prone to subjectivity, as it heavily relies on the prior experience of the clinician, resulting in higher inter-rater variability [19, 30, 64–66]. Therefore, adjunct quantitative measures of amyloid deposition and more sensitive thresholds are beneficial [25, 67–69]. In addition, quantification could hold a range of benefits and clinical utility on top of current binary classification, such as improvements in diagnostic confidence, prediction of cognitive decline, and changes to patient management [58, 70–74]. Similar utility has been shown for other neurological disorders, for example, quantification of regional atrophy patterns in dementia [75–78] and traumatic brain injury [79, 80]; hippocampal sclerosis and quantitative T2 signal in temporal lobe epilepsy [81–84]; stroke severity quantification by critical care physicians [85, 86]; pre-surgical planning and survival prediction in glioma resection [87, 88]; and lesion load measurements in multiple sclerosis [89–91]. The various quantitative measures available for amyloid PET quantification are discussed in detail later in the review.

#### Aims of this state-of-the-art review

In this review, methods for quantification of static amyloid PET scans are summarised and compared along with a discussion of the overall utility of amyloid PET quantification in routine clinical practice, observational research, and clinical trials. The general aim is to facilitate greater understanding and wider use of sensitive standardised methodologies for measuring Aβ pathology. More specifically, accurate cross-sectional and longitudinal measurement of brain amyloid pathology can support the use of amyloid PET biomarkers in clinical and research settings, by providing information on the extent of pathology. This could include the evaluation of both early and established amyloid pathology, improving our understanding of disease development, and consequently optimise individualised risk stratification. Full quantification using dynamic PET acquisition and determination of the non-displaceable binding potential (*BP*_ND_) were beyond the scope of this review; as such, the methods covered in this review constitute semi-quantification of amyloid PET. Indeed, factors such as acquisition time window and regional cerebral blood flow can impact methods based on static acquisitions, although the latter does not play a major role in an early AD population [92, 93]. For a review on the value of full PET quantitation, see Lammertsma [94].

## Quantitative measures for clinical assessment of amyloid burden

Quantification of static amyloid PET scans can be performed using software packages to calculate both regional and composite levels of amyloid burden. Importantly, these packages generate a continuous measure of amyloid burden which can be used in addition to dichotomous visual reads. Currently available measures are the more commonly used standardised uptake value ratio (SUVr) [95], the Centiloid (CL) scale [74, 96], and reference-based *z*-scores [97], while the more recent methods include the Aβ load [98], Aβ index [99], and AMYQ [100]. Both CL and *z*-scores are calculated based on SUVr, whereas the emerging methods use different approaches to select the target and reference regions for segmenting regions of interest (ROIs). In addition, each method provides a unique unit/scale and specific metric for quantification, which motivated inclusion in this review. A key area of current research focusses on the potential sensitivity of visual assessment and quantification methods to variation in scanners [101], reconstruction algorithms [102–104], scanning time, and scanning window [93, 105, 106], all of which can affect both visual assessment and quantification. See “Future directions” later in this review for an overview of ongoing technical validation studies. While these quantification measures are becoming increasingly common for research purposes, some of these metrics have also been used in clinical practice and trial settings. Quantification could supplement visual inspection of amyloid PET imaging, especially for (i) less experienced readers [63]; (ii) equivocal (“grey zone”) cases [107, 108] where diagnostic confidence is low [109]; and (iii) for assessing isolated regional uptake [57, 110]. In clinical trials, quantification can be used to better guide patient enrolment and for therapy response monitoring [111–114].

### Standardised uptake value ratio

**Fig. 2:**
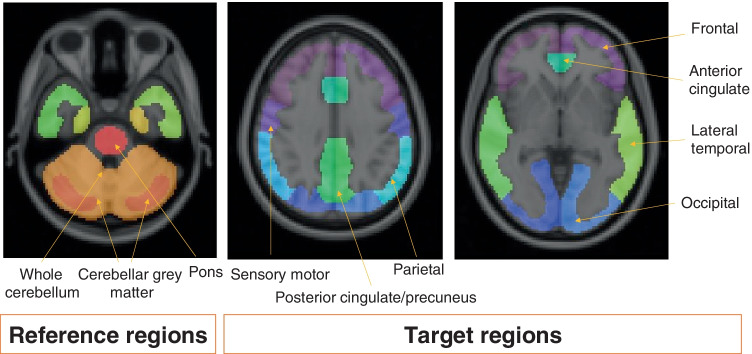
Example of the most common reference and target regions used when generating SUVr

The most widely used measure for quantifying amyloid burden is the SUVr. It is a simplified method based on computing the ratio of tracer uptake between a target region and a reference region in a late (static) PET acquisition, when the radiotracer is expected to have reached pseudo-equilibrium [95] (Fig. 2). Target regions can include either individual regions or be a composite of several (cortical) regions. Common ROIs in the amyloid PET radiotracer product labels include the medial orbital frontal cortex, anterior cingulate, lateral temporal lobes, precuneus, posterior cingulate, parietal lobe, and striatum. On the other hand, reference regions should ideally have no specific tracer binding, similar tissue characteristics/kinetics as the target regions, and tracer uptake in reference region should be unaffected by the disease under investigation, making the cerebellar cortex a suitable reference regions for amyloid tracers in most cases [94, 115]. Alternative reference regions have been proposed, such as the pons, whole cerebellum, and subcortical white matter, as their use generally results in increased stability of quantification over time [116–118].

Various software packages are clinically available (see “Regulatory approved tools and research methods for amyloid PET quantification” section below) to quantify brain amyloid using SUVr. Using these software approaches, optimal SUVr cut-offs for amyloid positivity have been defined using various approaches and for different purposes: receiver operating characteristics for differential diagnosis [119], comparison with histological findings [19, 20], and using young healthy adults as a definition for amyloid negativity [120].

SUVr is the most widely used and established metric [121], having been implemented in several recent trials to assess treatment efficacy [122–128]. However, accurate measurement and cut-off values are highly dependent on the chosen tracer, reference region (Fig. 2), and delineation method [74, 129, 130], which challenges the pooling of multi-centre SUVr data across tracers [131]. In addition, there is high variability in longitudinal results [93, 132], which limits the power in detecting genuine biological differences. SUVr values can also vary based on partial volume averaging effects [133, 134]. However, PVC intrinsically amplifies noise in trying to reduce bias and, therefore, a given PVC method needs to be finely tuned to the particular image characteristics so that the beneficial effects of the method outweigh anything detrimental.

### Centiloid scaling

As the use of different amyloid PET tracers grew in both clinical and research settings, there was a need for inter-tracer standardisation of the SUVr metric in multi-centre collaborations. To this end, the CL scale was developed [74], which is an unbounded 0 (mean grey matter signal of young healthy controls) to 100 (typical AD patient signal) scale that conveys a single patient’s amyloid burden based on two anchor points using the [^11^C]PiB SUVr from the Global Alzheimer’s Association Interactive Network (GAAIN) reference dataset (http://www.gaain.org/CL-project). The main aims of the CL scale were to (i) simplify and expedite direct comparison of Aβ PET results across sites and studies; (ii) outline the earliest thresholds for amyloid positivity and define the range of positivity in AD; (iii) robustly quantify longitudinal change; and (iv) facilitate inter-tracer comparisons [74]. Since then, several studies have tested the scale’s validity and used it to improve the harmonisation and standardisation of Aβ PET quantification across tracers, scanners, and analytical implementations [52, 96, 104, 118, 135–144].

The CL approach allows any site using amyloid PET to follow a multi-step process to generate a CL scaling from their own local Aβ PET data. The basic principle is to scale the ^18^F-labelled tracers’ SUVr to equivalent [^11^C]PiB SUVr, and this is further transformed to the 0–100 scale mentioned above. This process consists of a validation of the local pipeline using the GAAIN data and then the application to a new tracer [74, 138]. PET processing for CL quantification is often implemented through statistical parametric mapping (SPM) but other methods are available, including those without the use of an accompanying MRI [96, 145]. Routinely, PET images are first co-registered to their corresponding T1-weighted MR images and subsequently transformed to MNI space. Next, PET images are intensity normalised often using the whole cerebellum as the primary reference region, and other reference regions include pons, cerebellar grey matter, and whole cerebellum plus brainstem. Finally, CL values are generated using the mean values of the standard CL target region based on a previously calibrated transformation [74]. The team behind the CL project and producers of the approved fluorine-18 labelled radiotracers have made progress in deriving and verifying conversion formulae that enable translation of non-[^11^C]PiB Aβ PET semi-quantitative values to standardised [^11^C]PiB measures [52, 96, 136, 143], see Table 1 for conversion equations using the standard CL processing pipeline. However, please note that the CL method can be applied to any non-standard pipeline, thus leading to a potentially unlimited number of conversion equations.

**Table 1:** Conversion equations using the whole cerebellum as reference region applicable to the standard CL processing pipeline for generating CL scores with the most commonly used tracers, adapted from [101]

**Tracer**	**Variance (CL SD) young controls**	**Variance ratio (tracer SD/PiB SD)**	**Slope (tracer SUVR to PiB SUVR)**	**Intercept**	**R**^**2**^	**CL equation** **CL =**
**[**^**18**^**F]Florbetapir** [143]	12.0	4.6	0.54	0.5	0.89	175.4*SUVR_fbp_–182.3
**[**^**18**^**F]Flutemetamol** [52]	5.4	1.54	0.78	0.2	0.95	121.4*SUVR_flute_–121.2
**[**^**18**^**F]Florbetaben** [136]	6.8	1.96	0.61	0.4	0.96	153.4*SUVR_fbb_–154.9
**[**^**11**^**C]PiB** [138]	3.5	n/a	n/a	n/a	n/a	93.7*SUVR_pib_–94.6

#### Implementation

**Fig. 3:**
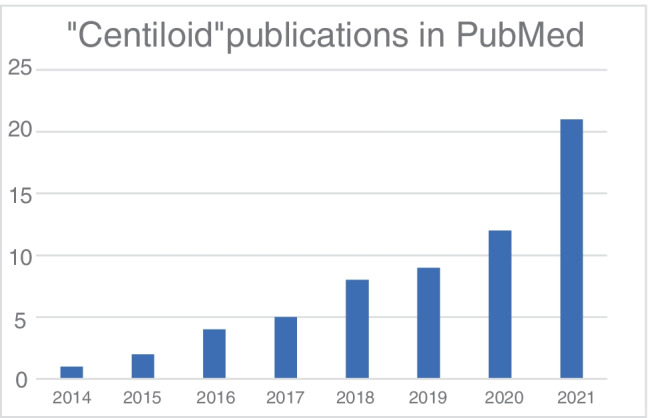
Bar graph showing the increasing use of CLs in academic publications. The numbers were obtained through a PubMed search for “Centiloid” in all fields on 7th September 2021

Since its development in 2015, the CL scale has been widely implemented in research studies, including both AMYPAD studies and various clinical trials (Fig. 3) [25, 51, 52, 57, 69, 107, 112–114, 132, 136, 138, 146–155].

One of the key advantages of an “absolute” metric of amyloid burden is generalisation of quantitative thresholds across tracers and pipeline implementations. Universal cut-off or threshold values to denote amyloid status can be applied alongside visual reads and in longitudinal multi-centre studies to facilitate inter-centre and inter-tracer comparisons. The CL approach has been validated against neuropathology [148, 149] where CL < 10 correlates with absence of neuritic plaques, CL > 20 specified at least moderate plaque density, and > 50 CL best confirmed both neuropathological and clinicopathological evidence of AD. Clinical studies have also validated thresholds for amyloid PET positive status [25, 132, 146], defined “grey zone” patient cut-offs [107] and derived CL cut-offs to detect early amyloid abnormalities in cognitively unimpaired individuals [69, 150–152]. Predictive models using the CL scale have been developed for calculating rate of cognitive decline in cognitively normal subjects [153–155]. In addition, Hanseeuw et al. [156] found that a CL threshold of 26 in memory clinic patients optimally predicts progression to dementia 6 years after PET.

**Fig. 4:**
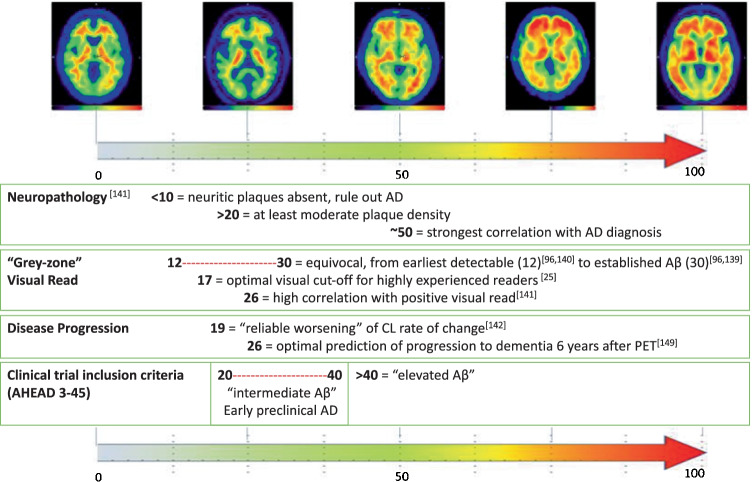
Summary of the various CL thresholds established in the literature and in use for clinical current clinical trial inclusion

In clinical trial settings, quantification may be used to identify the optimal window for therapeutic intervention [157]. This is illustrated by the AHEAD 3-45 study, which requires participants to have specific levels of amyloid pathology, either “intermediate” (20–40 CL) or “elevated” (> 40 CL), signifying the added value beyond binary classifications [158]. The CL scale has been used in clinical trial settings to track therapy response measure [111–114, 159, 160], determine strategies for reducing AD prevention trial sample sizes [161], and improve patient selection for trials [48, 162] and could assist in treatment endpoint decisions [51]. Various cut-offs established in the literature are summarised in Fig. 4.

### Z-scores

*Z*-scores represent the number of standard deviations from the mean of a reference or control group and are generally based on SUVr values. It can be calculated for both composite cortical regions, individual regions [97], and at voxel level [58, 163]. Therefore, *z*-scores are another method for establishing whether a subject’s amyloid deposition should be considered abnormal. Previous work using a classification threshold of *z* = 2.0 demonstrated high concordance with visual read and an autopsy cohort [97]. Based on a set of amyloid negative subjects, an average image (*NID*_*Ave*_) and a standard deviation image (*NID*_*SD*_) are created. The patient scan (*Pat*) is then compared to this reference database as follows:

$${Pat}_{Zscore}=\frac{Pat_{SUVR}-{NID}_{Ave}}{NID_{SD}}$$

#### Implementation

*Z*-scores are widely used in several areas of medical research. In the context of amyloid PET, four of the five commercial software packages covered in this review generate *z*-scores. One recent study compared the results of two packages with visual assessment, reporting that both software packages provide a high sensitivity and can assist with reporting more complex cases, such as those with atrophy or poor grey-white matter differentiation [164]. Optimal *z*-score thresholds for amyloid positivity have been established for the pons (1.97) and cerebellar cortex (2.41) as reference regions [165]. These thresholds have been validated against histopathological classification and visual read [97]. Several studies have used *z*-score maps for predicting and measuring temporal trajectories and patterns of Aβ and tau accumulation in AD [70], where the entorhinal cortex is flagged as one of earliest areas for tau deposition and medial cortical areas for Aβ deposits [166–168].

### Aβ load

With the aim of increasing sensitivity for detecting change and therefore statistical power in clinical trials, the Aβ load metric was developed by Invicro (https://invicro.com/case-studies/amyloid-load/) as a novel approach to quantify global Aβ burden using [^18^F]florbetapir as the test ligand. In line with CL, PET images are co-registered to a corresponding T1-weighted MRI and transformed to MNI space. The Aβ load metric is then generated based on spatiotemporal modelling work as a linear combination of two previously defined canonical images: (i) nonspecific binding of [^18^F]florbetapir and (ii) “Aβ carrying capacity,” which is the greatest possible Aβ concentration for a specific region [169]. The final Aβ load calculation is performed with the MATLAB-implemented “Amyloid^IQ^ algorithm,” which uses both cross-sectional and longitudinal PET and MRI from ADNI to generate a percentage of global Aβ burden [98, 169].

#### Implementation

The Aβ load metric has been implemented for assessing amyloid accumulation in Down’s syndrome [170, 171] and in a multisite analysis of the concordance of visual read and amyloid PET quantification [65], which found 92.5% concordance across 120 scans.

### Aβ index

The Aβ PET pathology accumulation index does not require an MRI as it is based on a PET-driven principal component analysis (PCA) method [99, 172]. The Aβ index corresponds to a weighting factor acquired during spatial normalisation of the images to MNI space using a previously described adaptive principal component template [172]. Two principal components are generated using the single value decomposition from SUVr images: (i) the average of the images and (ii) either the specific binding or the elements of discrepancy between Aβ positive and Aβ negative scans. A synthetic template is generated using the linear combination of these two principal components, from which a bounded metric between −1 and 1 is generated to define the global Aβ burden.

#### Implementation

The Aβ index has not been widely used to date. Nonetheless, it was recently used in a study comparing visual read and automated methods for amyloid PET processing, where an optimal cut-off score of −0.36 achieved a sensitivity of 97% based on visual read in 155 elderly controls over a 4.5 year follow up [173].

### AMYQ

The most recently developed technique is AMYQ, which is based on similar methodology to the Aβ index, does not require an MRI scan, and is interchangeable across tracers [100]. As with the Aβ index, a synthetic amyloid template is generated using PCA and is independent of predefined regions of minimal cortical load or corresponding reference regions for scaling the PET. AMYQ uses the same scale as CL and was recently validated against CL for detecting amyloid positivity (area under curve > 0.94) and for accuracy in differentiating AD dementia patients and controls [100]. AMYQ is yet to be used or validated in further clinical studies.

## Comparison of quantitative measures for assessing brain amyloid

**Fig. 5:**
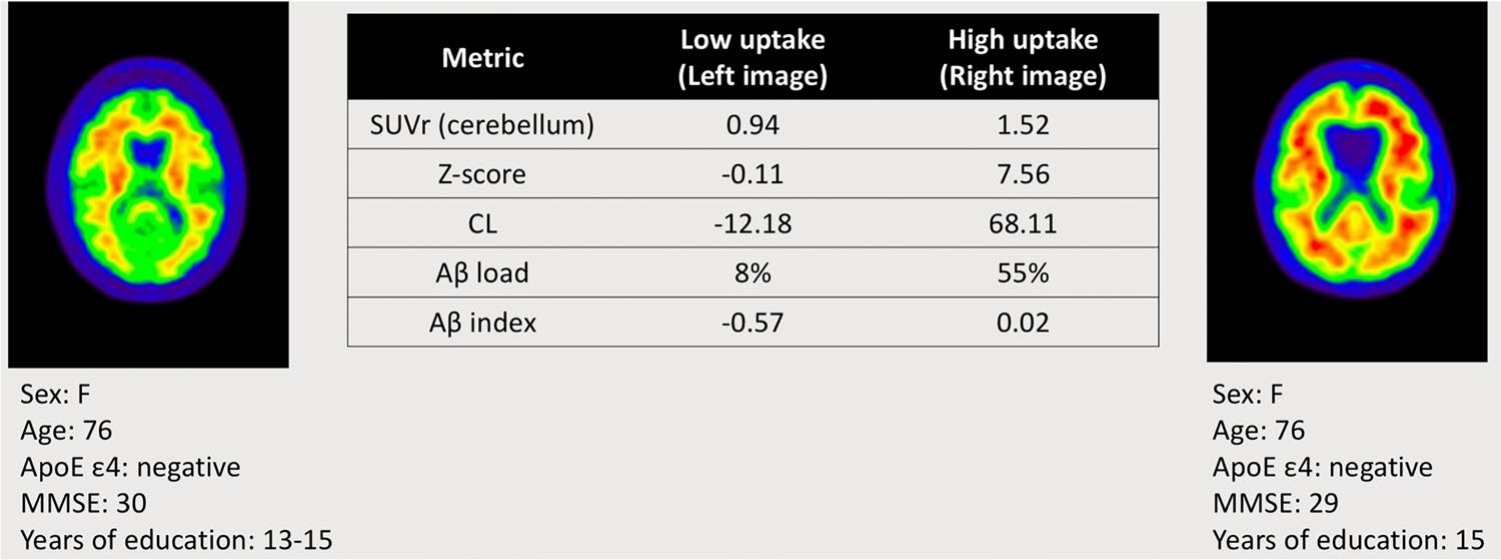
Example of quantitative metrics computed on two subjects from the AIBL dataset scanned with [^18^F]flutemetamol. Low amyloid uptake (left image) and high amyloid uptake (right image), including demographics. It was not possible to compute AMYQ due to the proprietary nature of the software. Abbreviations: mini-mental state examination (MMSE), standardised uptake value ratio (SUVr); amyloid-β (Aβ)

The various methods have been summarised for direct comparison in Table 2, and Fig. 5 shows an example of each measure calculated from a subject with high and one with low amyloid uptake.

**Table 2:** Comparison of the various methods available for amyloid PET quantification

Metric	Units	Basis of measure	Utility/widespread use	Validation	Imaging needs	Strengths	Weaknesses
SUVr	Ratio	Ratio of tracer uptake between a target and reference region	Widely implemented through CE/FDA-approved software	Versus visual read in controls, MCI, and AD patients [173–175] Test-retest [21, 64, 175, 176] Histopathology [19, 97] and CSF [177]	Static PET Structural MRI, although SUVr can be calculated PET only from template ROIs	Easy to calculate across multiple regions Available through CE/FDA-approved software Widely validated against other measures on a variety of clinical populations	Dependent on tracer, reference/target region, and analytical implementation Variability in longitudinal studies [116, 129], resulting in limited power for detecting biological differences
CL	Centiloids (0–100), unbounded	Mean amyloid deposition of young healthy controls (0) to typical AD patients (100)	Increasingly widely used in research and clinical settings, available through CE/FDA-approved software	Against SUVr, and including test-retest [74] Neuropathologically [148, 149, 178] and CSF [147] Positivity threshold validation [146]	Static PET Structural MRI recommended Needs to be calibrated via [^11^C]PiB or a surrogate reference tracer	Universal, tracer independent metric available through CE/FDA-approved software Widely validated against other measures on a variety of clinical populations Easily interpreted	MRI recommended, although more recent iterations have removed this requirement [96, 145] Currently only validated for global/whole brain ROIs rather than regional — not as sensitive to focal uptake as regional measures
*Z*-score	Standard deviations	Difference from mean of a cognitively healthy population	Widely implemented through CE/FDA-approved software	Widely validated statistical metric for amyloid positivity [58, 97, 109, 164, 179]	Static PET	Well known and widely used metric available through CE/FDA-approved software Easy to calculate across multiple regions, easily interpreted	Reliant on accurate SUVr measurements Dependent on reference/target region and analytical implementation Requires a normative reference database
Aβ load	%	Global Aβ burden	Not widely used	Against SUVr [98]	Static [^18^F]florbetapir PET Structural MRI	Larger effect sizes than SUVr — increased power in clinical trials Easily interpreted as a %	Unavailable through CE/FDA-approved software Tracer specific ([^18^F]florbetapir), although work is ongoing for other tracers Not yet widely validated Assumes spatially harmonised pattern of amyloid accumulation according to the maximum carrying capacity of each region
Aβ index	−1, 1	Global Aβ burden/specific binding	Not widely used	Against SUVr, CSF, visual read, and neuropathology [99, 173]	Static [^18^F]florbetapir and [^18^F]flutemetamol [^18^F]florbetaben work is ongoing [180]	Does not require an MRI Interchangeable across [^18^F]florbetapir and [^18^F]flutemetamol PET Independent of reference and target regions	Unavailable through CE/FDA-approved software, although planned to be incorporated as part of Hermes Medical Solutions’ *BRASS* software Not yet widely validated or implemented
AMYQ	0–100, unbounded	Global Aβ burden	Not widely used	Against CL and neuropathology [100]	Static PET	Does not require an MRI Interchangeable across tracers Independent of reference and target regions	Unavailable through CE/FDA-approved software Not yet widely validated or implemented

## Regulatory approved tools and research methods for amyloid PET quantification

Various regulatory approved (FDA 510k/CE-marked) software packages currently exist for automated quantification of amyloid PET:

1. Syntermed’s *NeuroQ* (https://www.syntermed.com/neuroq) — which generates *z*-scores and a “cortex-to-whole cerebellum ratio” based on the standard SUVr.
2. Hermes Medical Solutions’ *BRASS* (https://www.hermesmedical.com/neurology/) — which generates an SUVr relative to the whole cerebellum and a *z*-score (≥ 2 in ROI is considered positive) based on a normative database of 80 healthy controls [164].
3. MIM Software’s *MIMneuro* (https://www.mimsoftware.com/nuclear_medicine/mim_neuro) — which uses a standardised PET template registration to generate voxel-based surface projections, regional and mean SUVr, and *z*-score statistics without the need for an MRI [181]. MIM has also recently implemented the Centiloid scale across multiple tracers.
4. GE Healthcare’s *CortexID* (https://www.gehealthcare.com/courses/aw-cortex-id) — which generates SUVr and *z*-score surface projection maps of individual patients [97].
5. Siemens Healthineers’ *Syngo.VIA* (https://www.siemens-healthineers.com/medical-imaging-it/advanced-visualization-solutions/syngovia) — which uses positive cut-off value of SUVr ≥ 1.17 and a further threshold of SUVr ≥ 1.08 to assess “identifiable” levels of Aβ plaques, both of which are derived from previous work [182].
6. Qubiotech’s *Neurocloud PET* (https://www.qubiotech.com/en/solutions/neurocloud-pet/) — which generates individual SUVr using the whole cerebellum as a reference region.

Other tools are available in research settings, such as PMOD (https://www.pmod.com/web/?portfolio=CL), MIAKAT (https://nmmitools.org/2019/01/01/miakat/), CapAIBL [183], EvaLuation of Brain Amyloidosis (ELBA) [184], and NiftyPET (https://github.com/NiftyPET/NiftyPET) [102]. In addition, companies with CE/FDA cleared tools in the radiology AI space have software in development and validation stages, such as ADM diagnostics (https://admdx.com/), icometrix (https://icometrix.com/services), Cortechs.ai (https://www.cortechs.ai/products/petquant-2/), and Combinostics (www.combinostics.com). However, detailed coverage of these tools is beyond the scope of this review as they are not yet approved for clinical use.

## Why is amyloid PET quantification valuable and clinically beneficial?

In this review, various methods for automated quantification of amyloid PET measures are presented and discussed. These methods are becoming more widely available and there is a duty of care to the wider dementia community to increase their visibility and facilitate greater understanding of these methods.

### Quantification in clinical practice

Quantification of amyloid PET has shown strong concordance with binary visual assessment in several studies [25, 57, 63, 65, 66, 97, 107, 109, 173, 174, 185]. Amyloid PET tracers available in clinical and research settings have all demonstrated comparable cross-sectional amyloid SUVr results [186–188]. However, there has been no direct head-to-head comparison of the three tracers within the same cohort. As such, the individual effectiveness of each tracer in, for example, assessing an equivocal test set remains to be seen. Other recent studies have found that using quantification alongside visual reads improves diagnostic confidence [33, 36, 38–40, 189], accuracy, and consistency for (i) early detection of amyloid (mild AD, MCI, and controls) [58, 190]; (ii) less experienced readers, i.e. those with visual read accuracy of ≤ 90% [63]; and (iii) more difficult to interpret cases, such as patients with unclear diagnoses or weaker grey-white matter differentiation [57, 109, 164]. However, additional inter-tracer standardisation is required to facilitate multi-centre patient assessment, collaborations, and longitudinal evaluation [191]. More broadly, there remains a need to increase the general understanding of quantitative measures and their diagnostic information. SUVr is the most widely used metric but, as previously mentioned, accurate results are highly dependent on chosen reference region and its delineation [74, 129, 130]. The CL scale could provide a continuous standardised metric that aligns the use of target and reference regions and harmonises the outcome measures [74, 146]. Multiple standardised cut-offs have also been established to progress beyond simple binary stratification, provide prognostic information, and predict cognitive decline (Fig. 4).

Objective quantification of amyloid burden is imperative now more than ever due to the recent approval of aducanumab (Aduhelm) in the USA and the potential availability of other amyloid targeted therapies. Putting controversies aside, accurate Aβ measures are essential for prescribing the drug, and future similar drugs, most effectively in clinical practice. For example, prophylactic removal of amyloid may not be suitable for all amyloid positive patients, such as those with dual pathologies and mixed dementia [192]. The aducanumab appropriate use recommendations advocate determination of amyloid status but do not cover when, if ever, treatment should stop or the requirements of a maintenance regimen [193]. In the future, therapy response monitoring with quantitative metrics might be relevant from a perspective of patient burden and health economics. With further research, quantitative amyloid PET could provide universal thresholds alongside visual assessment for deeming treatment as either successful or unsuccessful on a per patient basis, and thereby assisting in the decision to continue or cease treatment. Further work on the clinical benefit of adjunct quantification is encouraged; see Table 3 in “Future directions” for an overview of relevant ongoing AMYPAD studies.

**Table 3:** Overview of current validation requirements in amyloid PET quantification and the associated AMYPAD studies currently underway

What research is still required to validate amyloid PET quantification?	What studies are in place to perform this validation?
**Technical validation**	
Measure agreement among quantification and visual read across cohorts to assess robustness across populations	Diagnostic and Patient Management Study (DPMS) [50] and Prognostic and Natural History Study (PNHS) [51]
Evaluate the utility and robustness of longitudinal quantification measures	Systematic review (*PROSPERO ID: CRD42021254695*) updating previous work by Schmidt et al., from 2015 [134]
Calculate the impact of data harmonisation on global CL quantification	Ongoing work presented at AAIC 2020: “Harmonization of Amyloid PET Scans Minimizes the Impact of Reconstruction Parameters on Centiloid Values” [103]
Assess CL stability as a function of pipeline design, reference region selection, cortical target, and image resolution. Provide optimal pipeline for multi-centre studies	Ongoing work presented at AAIC 2021: “Evaluating robustness of the Centiloid scale against variations in amyloid PET image resolution” [194]
Compare static acquisition derived metrics with full quantitation derived from dual-time window dynamic imaging	“Parametric imaging of dual-time window [^18^F]flutemetamol and [^18^F]florbetaben studies” [195]. Tertiary outcome of the PNHS; predicting disease progression analyses [51]
**Routine clinical use (diagnostic settings)**	
Determine clinical utility of amyloid PET quantification using a randomised-controlled trial design	Primary outcome of the DPMS [50]
Formally test if and when quantification approaches support visual assessment of difficult cases	Secondary outcome of the DPMS [50]
Assess the value of regional visual read and quantification in routine clinical settings	Tertiary outcome of the DPMS [50]
**Scientific and clinical trial settings**	
Assess value of quantification to improve risk stratification and individualised disease trajectory in the earliest stages of AD	Primary outcome of the PNHS [51]

### Quantification and prevention trials

In addition to clinical practice, established CL thresholds can also be used to improve clinical trial enrolment [48, 158, 161, 162], assess treatment response [111–114, 159, 160], and, as previously mentioned, potentially guide treatment endpoint decisions. Aducanumab is not an AD dementia panacea and will likely form part of a combined therapy [193, 196–198]. Indeed, there are several ongoing and planned clinical trials of novel anti-amyloid and anti-tau agents. These phase II and III trials are large, multi-centre and multi-tracer with the inclusion of data from different scanners, which have implemented standardised and validated quantitative metrics, such as the CL scale. Furthermore, in clinical trials of multiple active dose and placebo-controlled arms, PET signal changes must be averaged across subjects in each treatment arm, highlighting the value of the CL scale. Quantitative metrics will also be critical in establishing the ideal disease stage for therapeutic intervention and if/when to withdraw a drug [69, 161, 162, 199]. Trials are increasingly enrolling cognitively unimpaired individuals who have started to accumulate regional Aβ but are still considered “negative” both visually and dichotomously, i.e. preclinical AD [6, 200]. In these cases, visual reading can be challenging but quantification could automatically flag this “grey-zone” status [153–155, 190, 200].

### Regional measures of amyloid burden

Regional estimates of Aβ deposition measured with PET scanning are a potential advantage over CSF and blood-based biomarkers, which do not convey this valuable information [201]. Recently, the field of AD research has focussed on the value of the topographical distribution and extent of amyloid burden, beyond binary classification of the amyloid status [199, 202, 203]. Studies so far have demonstrated the added value of this information for both disease-modifying therapies [112] and in clinical use, especially during the earliest phases of amyloid accumulation where cognitive symptoms are subtle [38, 110, 204–206]. In these cases, regional assessment has improved detection and there remains a need to reliably quantify this early amyloid pathology as secondary prevention trials, such as the AHEAD 3-45 study, move to treat preclinical AD subjects with low but detectable Aβ levels [200]. Additionally, there is benefit in improving the prognostic value of amyloid imaging in routine clinical practice, by considering the regional location and extent of pathological load, which could improve subject placement along the AD trajectory [199, 207, 208]. While useful, regional quantification brings an additional challenge where smaller regions are more sensitive to quantification errors and confounding factors, such as partial volume effects and changes in cerebral blood flow.

### Possible influence of cerebral blood flow

Quantitative measures remain sensitive to changes in cerebral blood flow (CBF), albeit less of an issue in early stages of dementia [92, 93]. This may reduce the accuracy of longitudinal assessment [134] and acquisitions outside of the predefined time window. This review is broadly targeted to the generalist reader rather than specialists but it is worth noting that other (fully) quantitative approaches do exist. These methods require dynamic PET acquisitions and pharmacokinetic modelling using a plasma or reference tissue input. From these scans, the specific tracer binding can be derived, as changes in physiological factors are accounted for, such as CBF and tracer clearance [93]. However, these measures face a similar dependency on radiotracer and also require a longer dynamic acquisition protocol with complex processing requirements, which limits routine clinical use. Future longitudinal intervention studies could make greater use of dynamic imaging to measure smaller effects but this is much less likely in clinical routine due to time constraints [161]. Dual-phase or dual-time window protocols could be considered instead, as they provide measures of specific tracer binding but with shorter acquisition protocols [105, 106]. Nevertheless, the gain in precision would need to be beneficial to the overall workflow and should not supersede routine scanning otherwise.

## Future directions

Across the field, there are several initiatives aiming to assess the direct impact of amyloid PET, both clinically and in terms of health economics. While large projects such as the IDEAS trial and ABIDE study [33] already demonstrated the substantial effect on diagnosis and patient management, more recent outcomes are focussed on how undergoing amyloid PET affects hospitalisation, and therefore medical costs. In addition, differences among racial and ethnic groups are under investigation in the Health & Aging Brain among Latino Elders (HABLE) [209]. The next IDEAS phase aims to address racial disparities by recruiting a diverse cohort of at least 2,000 African American and 2,000 Latino subjects among the planned study population of 7,000 [210]. The IDEAS team recently published their PET-only processing pipeline to support the use of standardised quantitative measures in heterogeneous datasets [211]. These efforts are paramount to optimising the use of amyloid PET quantification in clinical routine and trial settings.

Within this context, the AMYPAD initiative covers several projects on the utility, robustness, and harmonisation of amyloid PET, especially for longitudinal measurements. As a body of work, the planned and current studies encompass the relevant validation necessary to drive greater uptake of quantitative measures in clinic for the benefit of patients worldwide. The ongoing AMYPAD studies aiming to meet these validation requirements are also outlined in Table 3.

Although it is a topic beyond the scope of this paper, quantitative analysis is likely to be complemented by AI-driven analysis techniques in the future. Indeed, various deep learning-based strategies currently exist for amyloid status prediction [212, 213] and SUVr quantification [214], and it will be of great interest to see how techniques such as these develop and contribute to the field.

## Limitations

Given that this review focusses on the clinical utility of amyloid PET quantification, it was out of scope to assess amyloid PET vs CSF or plasma amyloid measures, other experimental tracers, or PET imaging measures of neuroinflammation and synaptic density. While the CL scale has been used to assess amyloid and tau PET relationships and their prognostic value [215–217], discussion of tau PET was also out of scope although it remains a topic of interest. Furthermore, dynamic PET scanning can provide greater precision over static PET but requires longer acquisition time, which limits clinical use, and the overall added value still needs to be determined in different indications. As such, dynamic imaging protocols have not been fully discussed in this review. Finally, it was not possible to compute the AMYQ metric due to the proprietary nature of the software.

## Conclusion

In conclusion, several metrics are available to facilitate amyloid PET quantification. Accurate, tracer-independent measurements are needed now more than ever, and use of these methods is increasing. Individual strengths and weaknesses have been presented in this state-of-the-art review. Various recent methods do not require an MRI or a priori reference regions but they do require further validation in multi-centre studies against expert visual rating. The CL method has been widely validated and provides the dementia field with a continuous and universal metric. This method aligns the use of target and reference regions and harmonises the outcome measures. Several studies have validated CL thresholds for capturing the dynamic transition of patients from amyloid negativity to positivity, as well as for measuring disease progression, patient stratification, and prognostic assessment. However, further work is still required to determine threshold validity for longitudinal assessment, treatment endpoint decisions, clinical trial inclusion, optimising therapy intervention time points, and guiding dose selection.

## Data Availability

Not applicable for this review.

## Abbreviations

AD: Alzheimer’s disease
ADNI: Alzheimer’s Disease Neuroimaging Initiative
Aβ: Amyloid-β
A/T/N: Amyloid/tau/neurodegeneration
AIBL: Australian Imaging, Biomarkers and Lifestyle
CL: Centiloid
CBF: Cerebral blood flow
CSF: Cerebrospinal fluid
FDA: Food and Drug Administration
EMA: European Medicines Authority
GAAIN: Global Alzheimer’s Association Interactive Network
MCI: Mild cognitive impairment
PET: Positron emission tomography
PCA: Principal component analysis
PNHS: Prognostic and natural history study
SUVr: Standardised uptake value ratio
SPM: Statistical parametric mapping
SCD: Subjective cognitive decline
